# Riverscape dynamics and habitat utilization structure evolutionary diversification in a clade of Amazonian electric fishes

**DOI:** 10.1038/s41598-025-26512-0

**Published:** 2025-11-27

**Authors:** Jonathan G. Allen, Victor A. Tagliacollo, James S. Albert

**Affiliations:** 1 Department of Biology, University of Louisiana at Lafayette, Lafayette, LA 70503 USA; 2 Institute of Biology, Federal University of Uberlândia, Minas Gerais, Brazil

**Keywords:** Amazon, Dispersal, Extinction, Fishes, Hydrology, River capture, Speciation, Ecology, Ecology, Zoology

## Abstract

Rivers have long been implicated in the processes of macroevolutionary diversification, but only recently have tools emerged to quantify habitat volume and connectivity across modern and ancient landscapes. Here we compare biodiversity patterns in a diverse clade of Amazonian electric fishes with the predictions of three alternative hypotheses of rivers as: (1) semi-permeable dispersal barriers, (2) branching drainage networks, and (3) dynamic with a reticulated history of connections; i.e., river capture. We found support for all three hypotheses, with large river corridors as partial dispersal barriers to small-river species, interfluves as barriers to large-river species, and contrasting patterns of local (alpha) diversity and species-turnover (beta diversity) in large and small rivers. River captures are faster in smaller rivers with rare but expansive mega-river capture events, facilitating dispersal of small-river clades across watersheds. These results support the role of riverine dynamics as principle agents driving continental diversification of megadiverse tropical aquatic faunas.

**Supplementary Information:**

The online version contains supplementary material available at 10.1038/s41598-025-26512-0.

## Introduction

Biodiversity is distributed highly unevenly across Earth’s surface, with most described species inhabiting continental ecosystems^1^ and approximately 10% of all species restricted to continental freshwaters^2^. This high freshwater biodiversity is striking given that freshwater habitats occupy less than 1% of the Earth’s surface and an even smaller fraction (0.0001%) of its water volume^3^. Known as the “freshwater paradox,” this phenomenon is especially pronounced in vertebrates, in which over 25% of all described species are freshwater fishes^4^. An uneven distribution is also observed among river basins globally with about 10% of all fish species inhabiting the rivers, lakes and wetlands of Greater Amazonia^5^. This extraordinary biodiversity has drawn extensive theoretical and empirical attention^5–8^.

Rivers are hypothesized to shape freshwater biodiversity through their effects on dispersal, speciation, extinction, and adaptation^9,10^. While the ecological role of rivers in sustaining tropical lowland (< 250–300 m elevation) ecosystems is well established, their longer-term evolutionary impacts are less understood^9^. Recent work highlights how riverine and interfluvial barriers restrict gene flow, how stream size and connectivity structure local assemblages, and how river capture events create new opportunities for dispersal and isolation^6,10–12^. Documenting how these hydrological processes affect the ecology and evolution of aquatic biodiversity is increasingly urgent under the accelerated pace of anthropogenic changes to riverscapes^13^.

**Fig. 1:**
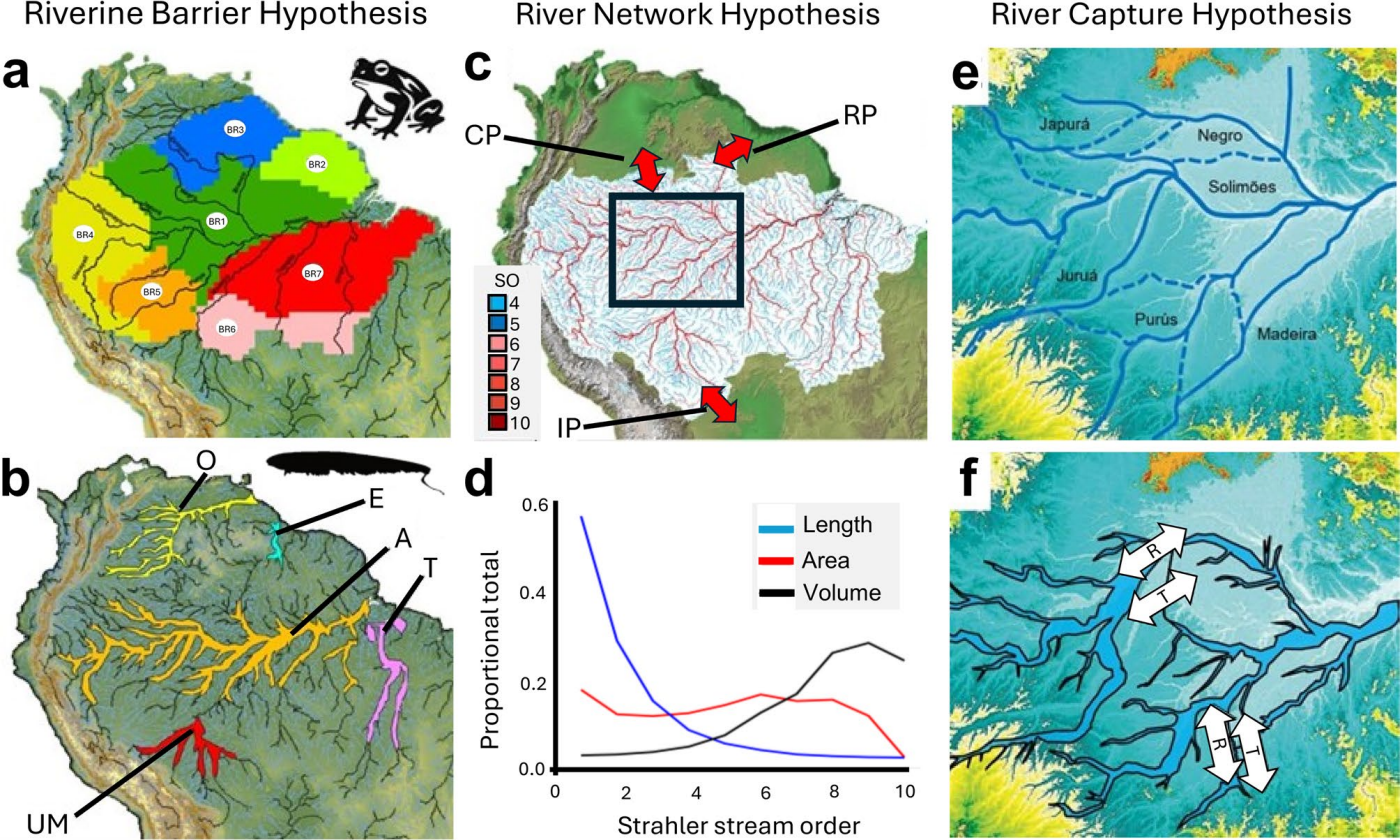
Three hypotheses on the role of rivers affecting diversification in Amazonian taxa. Hypotheses arranged left to right in order of increasing complexity, as minimum number of assumed parameters (see "Introduction"). (**a**) *Riverine Barrier Hypothesis* (RBH). Large rivers as semipermeable barriers to dispersal and gene flow. Example from *terra firme* adapted anuran amphibians^14^ (**b**) *Interfluvial Barrier Hypothesis* (IBH). *Terra firm regions* (green) between large rivers (black lines) as semipermeable barriers to dispersal and gene Example from riverine-adapted apteronotid electric fishes. Basin abbreviations: A, Amazon; E, Essequibo; O, Orinoco; T, Tocantins; UM, Upper Madeira. (**c**) *River Network Hypothesis*. Amazonian rivers by Strahler stream order (SO). Biogeographic portals between Amazonian tributaries and adjacent basins as double-headed arrows. CP, Casiquiare Portal; RP, Rupununi Portal; IP, Izozog Portal. Data from HydroSHEDS. (**d**) Proportions of river segments by SO for length, area and volume. (**e**) *River Capture Hypothesi****s***. Major reticulations of large rivers in the Central Amazon. Solid blue lines are current *thalwegs*; dashed lines paleo-thalwegs. (**f**) Large river corridors (dark blue) of the Central Amazon during the middle Pleistocene^15^. Note how river capture produces a reticulated history of connections among riverine (R) and *terra firme* (T) areas as biogeographic barriers and corridors. Maps created in ArcGIS Pro v.3.2.2 (Esri, Redlands, CA, USA; http://www.esri.com). Panel (**a**) modified from^14^ https://creativecommons.org/licenses/by/4.0/.

Three non-exclusive hypotheses are here proposed to explain how rivers contribute to evolutionary diversification, each emphasizing ecological processes at different spatiotemporal scales, and each making a distinct set of predictions regarding biodiversity patterns among taxa and areas (Fig. 1, Table 1). The ***Riverine Barrier Hypothesis*** (RBH) posits that large rivers function as barriers to dispersal and gene flow leading to genetic isolation and allopatric speciation^16^. The RBH was originally formulated to explain isolation and endemism among non-riverine (i.e., *terra firme*) taxa like primates^17^ and birds^18^. The ***Interfluvial Barrier Hypothesis*** (IBH) is similar to the RBH for obligate riverine species where interfluvial areas between riverine corridors act as barriers. Under this framework, both fluvial (i.e., channels and floodplains) and interfluvial (i.e., *terra firme*) areas can serve as semipermeable dispersal filters. The RBH and IBH have been implicated in many studies of plants, fishes, amphibians, reptiles, birds, and mammals (Table S1).

**Table 1:** Signature predictions and results of alternative hypotheses examined in this study: Riverine Barrier Hypothesis (RBH), Interfluvial Barrier Hypothesis (IBH), River Network Hypothesis (RNH), River Capture Hypothesis (RCH).

Hypothesis	Predictions	Examples
RBH	Riverine barriers more permeable to TF and eurytopic taxa upstream than DC taxa; Rank geographic ranges: TF and eurytopic taxa > DC taxa	*A. leptorhynchus* vs. *A. albifrons* vs. *P. hasemani* (Table S7)
IBH	Interfluvial barriers more permeable to eurtopic than stenotopic DC or stenotopic TF taxa; eurytopic species with larger geographic ranges	*"A." bonapartii* vs. *P. gimbeli* (Table S7)
RNH	Opposite alpha and beta diversity gradients along river continuum. TF rivers with low alpha and high beta; DC rivers with high alpha and low beta	Figure 3
RCH	River captures more frequent among TF rivers; δTF > δDC among TF rivers	δApteronotini > δNavajini among TF rivers (Fig. 4)
RCH	River captures less frequent among large DC rivers; δTF < δDC among large DC rivers	δApteronotini < δNavajini among large river basins; e.g. Amazon, Essequibo, Orinoco, Upper Madeira (Fig. 4)

Predictions and examples from this study. Strahler stream order (SO), Terra Firme (TF) in SO1-5, Deep Channel (DC) in SO6-10, eurytopic in SO1-10. Macroevolutionary dispersal rate (δ).

The ***River Network Hypothesis*** (RNH) posits that the dendritic geometry of river drainage basins shapes species composition and connectivity, influencing regional diversification^10,19^(Fig. 1B). Assessed by stream length, most Amazonian waterways are small rivers and streams with low Strahler stream orders (SO 1–5), including numerous geographically isolated headwater and lateral tributaries^20^ resulting in many localized assemblages with low alpha diversity (i.e., local species richness) and high beta diversity (i.e., spatial turnover in species richness between habitats or areas). By contrast, large rivers with high stream orders (SO 6–10) account for most Amazonian aquatic habitat by volume, are highly connected across wide geographic areas^21^, and exhibit high alpha and low beta diversity^22^(Fig. 1C, D). These contrasting diversity patterns along the stream order hierarchy are here hypothesized to contribute to regional diversification, with large rivers supporting many sympatric, syntopic and geographically widespread species, and isolated forest streams fostering rapid speciation and extinction.

The ***River Capture Hypothesis*** (RCH) posits that river network rearrangements drive diversification in obligate riverine taxa, by separating and merging river segments, increasing opportunities for speciation and dispersal respectively, and also increasing and decreasing geographic area and extinction risks respectively^23^. The RCH makes specific predictions regarding all three processes of macroevolutionary diversification, for more species on low-relief (topographically flat) lowlands, more endemic species on high-relief (topographically rough) uplands^6,23^, and higher rates of speciation and extinction (i.e. species turnover) in more connected portions of a drainage network that have faster rates of lateral-tributary transfer^24^. Among geological provinces of the world, low-relief areas include structural basins and areas of extended crust, whereas high-relief areas include upland shields, platforms and orogens^6^. The RCH is the most complex of the three hypotheses evaluated here, extending the effects of spatial heterogeneity on diversification expressed in the RBH and RNH into the time domain (Fig. 1E). The RCH predicts complementary patterns of geographic separation and merging among areas of riverine and *terra firme* habitat (Fig. 1F). The RCH incorporates the rate of change in connectivity by stream order where small river captures occur stochastically faster than larger captures^6,25^.

**Fig. 2:**
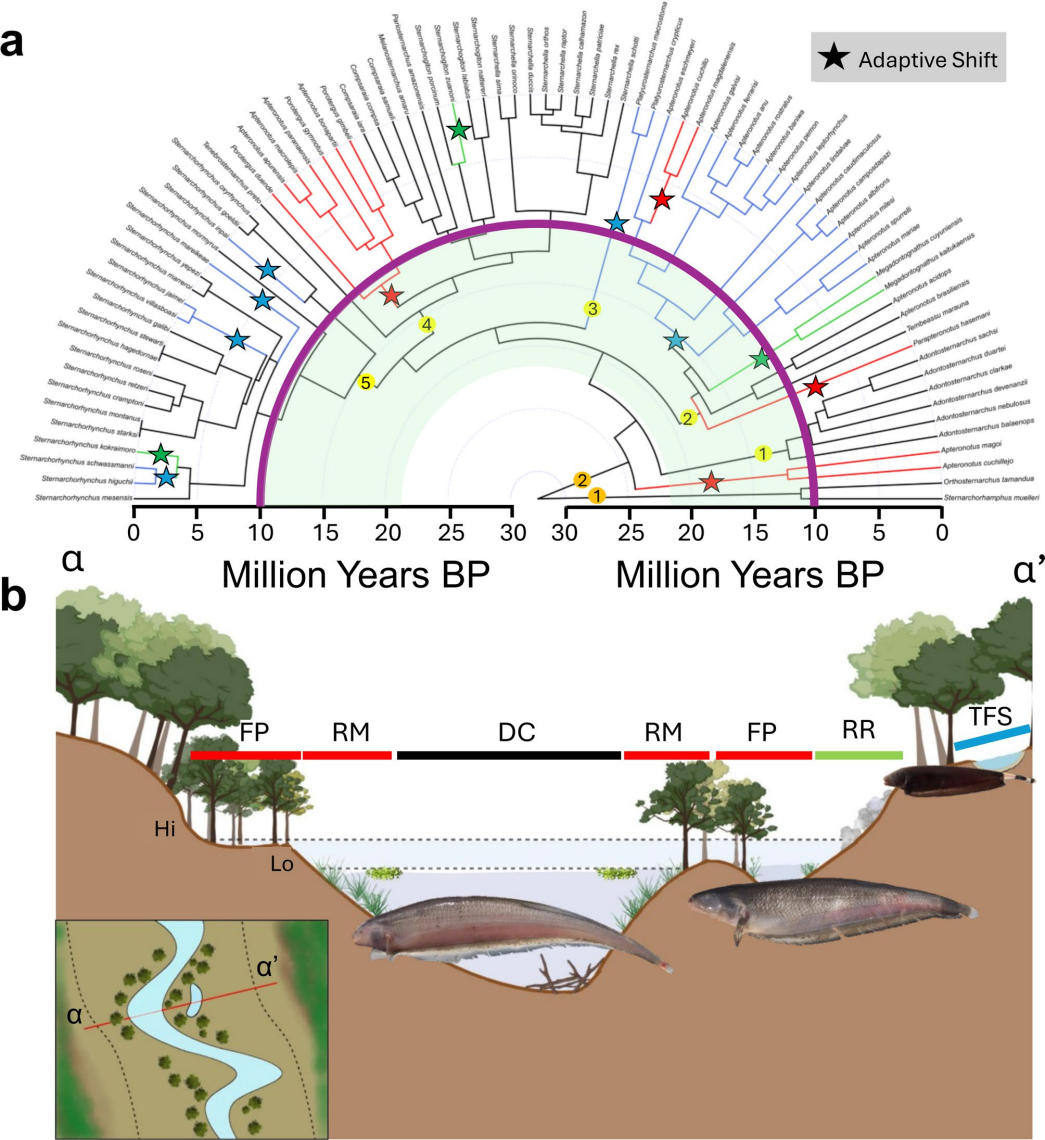
Habitat evolution in apteronotid electric fishes. (**a**) Time-calibrated phylogeny tracing evolution of adult modal habitat utilization (See SI text). Ecotrait data^30^; see Table S3. Habitats: DC = Deep Channels, RM = River Margins, FP = Floodplains, RR = Riffles and Rapids, TFS = *Terra Firme* streams. Purple line = origin modern Amazon c. 10 Ma. Shaded green = Pebas Megawetland c. 22—10 Ma. Star = Adaptive shift to habitat indicated by color. Note 13/163 nodes (8%) with adaptive shifts among these five habitats, indicating 92% functional redundancy. Orange circles (subfamilies): 1 = Sternarchorhamphinae, 2 = Apteronotinae. Yellow circles (tribes): 1 = Adontosternarchini, 2 = Apteronotini, 3 = Platyurosternarchini, 4 = Navajini, 5 = Sternarchorhynchini. (**b**) Schematic cross section of an Amazonian River floodplain illustrating habitats at high (Hi) and low (Lo) water with characteristic gymnotiform species; *Apteronotus albifrons* in TF; “*Porotergus” bonapartii* in FP; *Sternarchogiton nattereri* in DC. Inset: transect from α a to α’, floodplain margins as dashed lines. Note many apteronotid fishes use floodplain forests and river margins at high water but are constrained to river channels at low water. Created in BioRender. Allen, J. (2025) https://BioRender.com/wwad7x3.

**Fig. 3:**
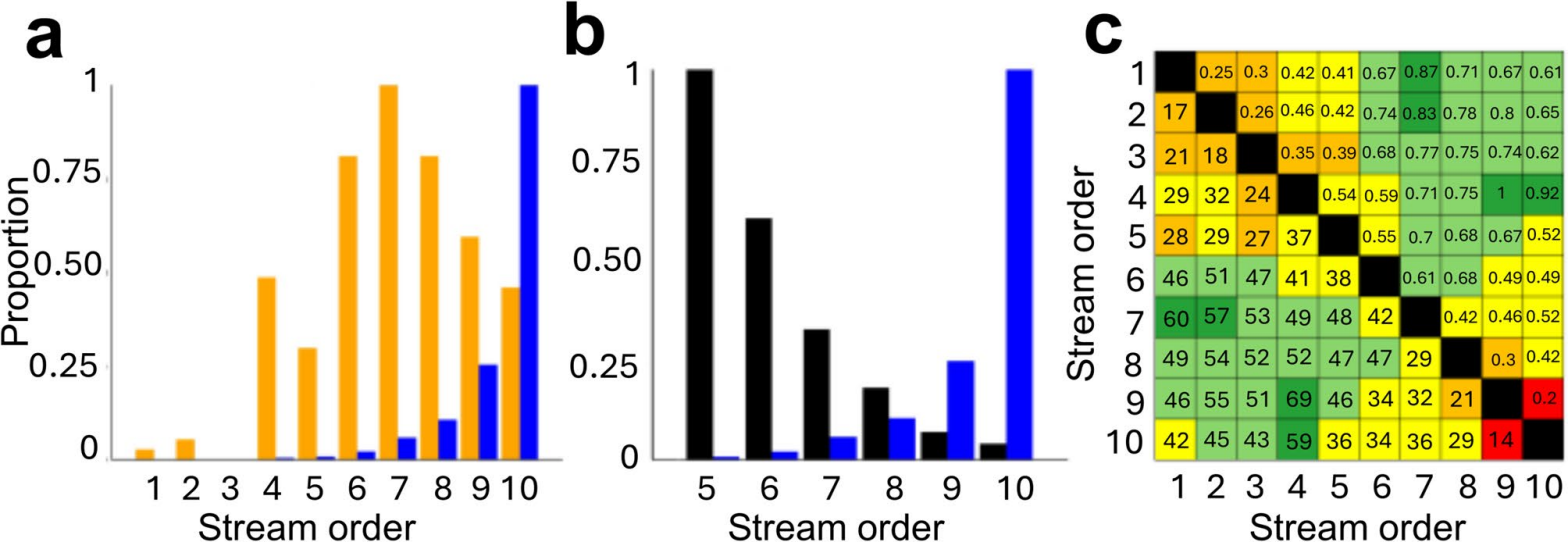
Diversity assessments for apteronotids by stream order support the Riverine Network Hypothesis. (**a**) Alpha diversity by SO. Orange = alpha diversity. Blue = alpha diversity/river length (km); (R^2^ = 0.45,* p* = 0.03). (**b**) Proportional diversity by SO. Black = beta diversity ${(\beta }_{W})$. Blue = alpha diversity / river length (km). (**c**) Pairwise species turnover matrix of ${\beta }_{A}$ values by river segments. Beta diversity values below and proportional values above diagonal. Note in A highest alpha in SO7 and highest proportional alpha in SO10, in B highest beta in SO5, and in C highest species turnover among SO between medium and largest rivers (SO 4 and 9), and most shared species in largest rivers.

Here we use empirical data from a clade of South American electric fishes (Apteronotidae: Gymnotiformes) to test these three hypotheses on the role of riverscape geometry and habitat utilization in structuring the evolutionary diversification of Amazonian fishes (Table 1). Apteronotidae was selected because it exhibits many biodiversity patterns typically observed in Amazonian fishes; i.e., high biomass and species richness in large, deep, lowland river channels, high species endemism in geographically isolated headwater tributaries of the upland Brazilian and Guiana Shields, ancient origins during the Oligocene (c. 30-35 Ma), and a broad geographic distribution across river basins of tropical South America with both cis- and trans-Andean species and clades^10,23^. Apteronotidae is a diverse taxon with about 100 described species in which most sister-species pairs have allopatric distributions, the species exhibit ecophysiological tolerances to distinct habitat types (e.g. small upland streams, floodplains, river channels, rapids), and the species are geographically distributed in a core-periphery pattern with high species richness in central Amazonia and high species endemism in the continental periphery^10,26^. We combine data from a species-dense time-calibrated phylogeny with newly available ecophysiological trait (i.e. ecotrait) data for all species, quantitative spatial data with stream order for c. 1.6 million river network segments, and paleogeographic data on river capture events during the Neogene (c. 22 - 2.6 Ma) hypothesized to have strongly affected evolution of the Amazonian biota^19,22,23,25,27^ (Figs. 2A,B, 3A,B).

Results provide partial support for all three of the hypotheses evaluated. Many (44 of 92 or 47%) apteronotid sister species pairs exhibit allopatric distributions across interfluvial watershed divides, and most (55 of 92 or 59%) apteronotid species exhibit a wide range of ecophysiological tolerances across stream orders (inhabiting more than three SOs). Most apteronotids exhibit phylogenetic niche conservatism with only 39 of 92 or 42% branches of the phylogenetic tree exhibiting shifts to different habitats or diets, and only two subclades evolving to inhabit smaller rivers and then dispersing to basins of the continental periphery by means of rare megariver capture events [*sensu* 6]. This unique combination of geographic conditions and organismal factors generated a biodiversity profile observed in many Amazonian fishes, with high local richness (*alpha* diversity), and high species turnover among habitats and river basins (*beta* diversity), patterns that result in high local functional diversity and high local functional redundancy^10,28,29^.

## Results

### Geographic and ecological distributions

Apteronotidae exhibits highest species richness in large (SO 6-10), deep (to 100 m) lowland (<250 m elevation) river channels of the Amazon and Orinoco basins, which is also the estimated ancestral habitat for this clade (Fig. 2). The two species of the subfamily Sternarchorhamphinae are entirely restricted to this habitat, and this is the most species-rich habitat for subfamily Apteronotini with 38 of 92 (41%) of the species (Fig. S2). Species richness values of the five tribe-level clades of Apteronotinae range over about one order of magnitude, from two species in Platyurosternarchini to 26 species in Navajini. All six species of Adontosternarchini are specialized midwater planktivorous fishes that inhabit large lowland river channels and floodplain lakes.

Apteronotini with 23 species has estimated origins in large lowland Amazonian rivers, but exhibits greatest species richness in other habitats, including 17 species in small to mid-sized rivers and streams (SO 1-5), two species (assigned to the genus *Megadontognathus*) in river rapids, and three species specialized for floodplains. Members of four Apteronotini subclades (i.e., *A. albifrons* clade, *A. brasiliensis* clade, *A. leptorhynchus* clade, and *A. magdalenensis* clades) have dispersed beyond Greater Amazonia into trans-Andean Maracaibo, Magdalena, and Pacific Coast river basins of Venezuela, Colombia and Panama, and to the La Plata (i.e., Paraguay and Paraná) river basins of Argentina, Paraguay, and Brazil, and the São Francisco river basin of Brazil (Fig. S2C).

Navajini exhibits greatest species richness in large and deep Amazonian river channels (with 18 species), and lower diversity in other habitats like river margins and smaller rivers (SO-3-5; eight species) and river rapids (one species; Fig. S2D). Members of two Navajini subclades have dispersed beyond the Amazon and Orinoco basins to the Essequibo basin of the coastal Guianas and the La Plata river basin. Sternarchorhynchini with 21 species exhibits a broad range of habitat tolerances geographic ranges across Greater Amazonia, with species inhabiting a wide range of stream orders and river water chemistry profiles. Members of at least two Sternarchorhynchini subclades have dispersed beyond the Amazon and Orinoco basins to the river basins of the coastal Guianas.

### River metrics

Following Horton’s laws describing the geometry and structure of river drainage networks, the stream lengths of South American waterways are dominated by small streams, with approximately 97% of total river length in lower-order streams and smaller rivers (SO 1–5), with only 3% flowing through higher-order rivers (SO 6–10; Fig. S1). In contrast, total aquatic volume increases dramatically downstream, with 90% of total river water volume concentrated in larger rivers. River surface area peaks at intermediate stream orders, reflecting a tradeoffs between stream length and habitat volume by stream order. Despite some regional variation, nearly all basins follow an inverse power function distribution of river length by stream order, with >95% of length in SO 1–4. Most basins also exhibit their highest river volume in large rivers (SO 6–10) with exceptions in the Maracaibo, Caribbean, and North-West Pacific basins, where volume peaks in smaller streams. These hydrological patterns are consistent across river basins but vary in magnitude. The Amazon basin alone accounts for 45% of the continent’s total river length. Smaller basins exhibit more surface area concentrated in the smallest streams, while larger systems such as the Amazon, Paraná–Paraguay, and Orinoco have proportionally more surface area and volume in higher-order channels.

### Biodiversity metrics

Apteronotid alpha diversity is lowest in small rivers and streams (SO 1-5) and highest in large rivers (SO 6-10) (Fig. 3A, Table S4). Normalizing diversity metrics by proportion of total river length shows a strong correlation between alpha diversity and stream order among all apteronotids (R^2^ = 0.45,* P* = 0.03; Fig. 3A). By contrast, Whittaker beta diversity values show highest turnover in species composition among mid-sized rivers (SO 5-6) and lowest turnover among largest rivers (SO 7-10) (Fig. 3B). Analysis of species turnover between all stream orders show that the largest river segments (SO 9 and 10) have the highest similarity in species composition (Fig. 3C). There are high similarity values at extreme ends of stream order continuum (e.g. SO1-2, SO9-10) and low similarity values for stream segments with intermediate stream orders (e.g., SO5-6) indicating that the number of endemics is higher in smaller stream orders and that largest rivers share the most number of species.

### Biogeographic analyses

**Fig. 4:**
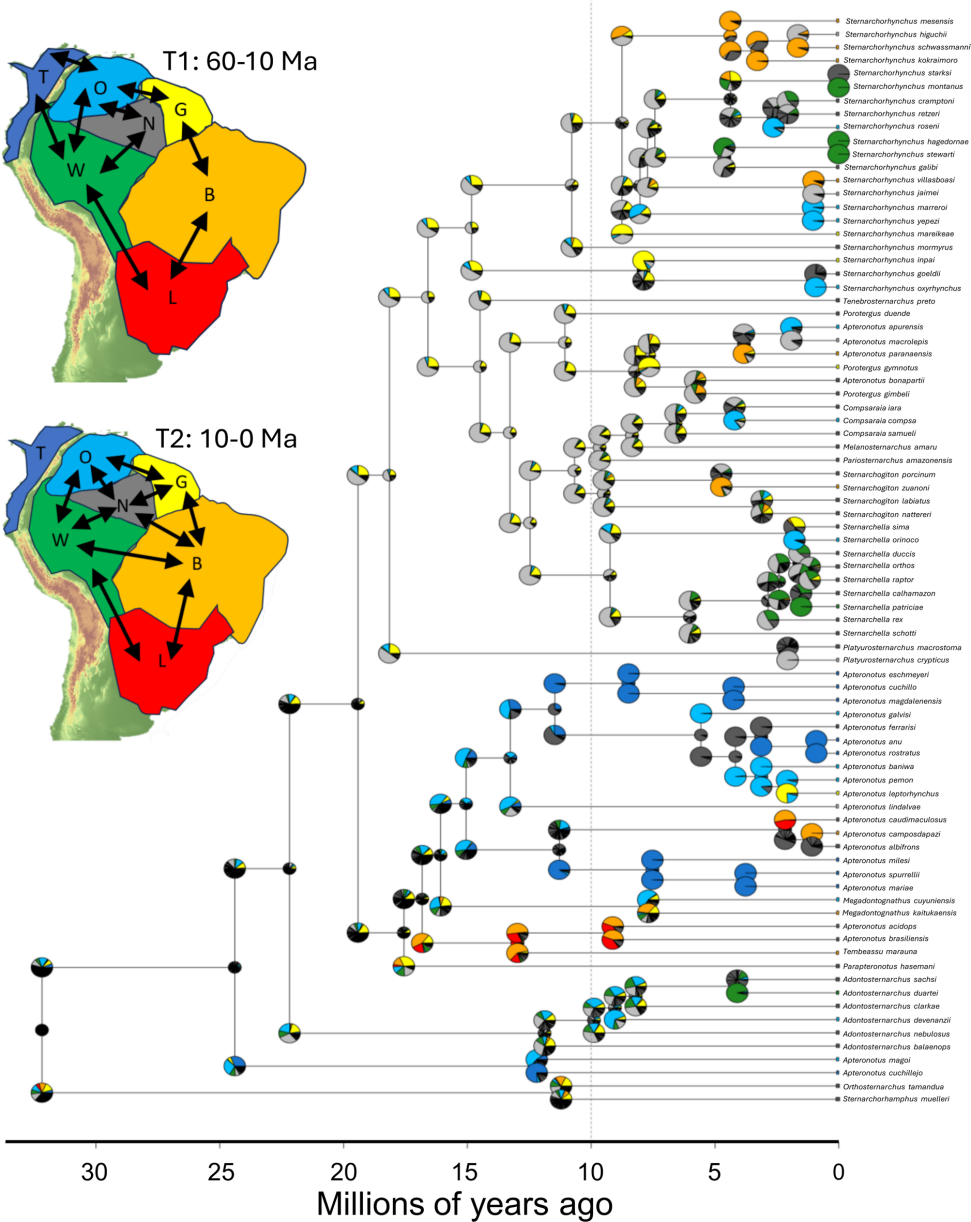
Ancestral Area Estimation of Apteronotidae using a two time-step paleogeographic model of bioregion connectivity (LEM1b). Time-calibrated phylogeny from Tagliacollo et al. (2024). Pie charts represent marginal likelihoods of ancestral areas using the DEC model of geographic range evolution in BioGeoBEARS. Time slices: T1: 30—10 Ma; T2: 10 – 0 Ma. Bioregions in inset; B = Brazilian Shield, G = Guiana Shield, L = La Plata, N = Negro, O = Orinoco, T = Trans-Andean, W = Western Amazon. Macroevolutionary dispersal rate (δ) 0.028 in Apteronotini, and 0.082 in Navajini (Table 2).

Biogeographic analyses using *Ancestral Area Estimation* (AAE) provided partial support for the role of paleogeographic events and paleoenvironmental conditions in structuring the formation of modern apteronotid diversity and distributions. Using *Akaike Information Criterion* (AIC) scores to evaluate model performance, the best-fit *Landscape Evolution Model* (LEM) for estimating contemporary patterns of biodiversity and biogeography in Apteronotidae was LEM1b, a two time-step model of bioregion connectivity based on documented paleogeographic hydrological connections (i.e. Amazon-Caribbean, Amazon-Orinoco, and Amazon-Paraná) (10, 19, 25, 31, 32). These changing connections through time resulted in geographic separations (vicariance and speciation) and connections (geodispersal and biotic dispersal) among adjacent sedimentary basins along the N-S axis of the Sub-Andean foreland basin and W-E axis of the Amazonian intracratonic basins (Table 2, Fig. 4).

**Table 2:** Maximum likelihood estimates and model comparisons for each landscape evolution models (LEM) pair.

LEM	Groups	Model Type	Species	# Regions	GRE	ln *L*	*K*	*δ*	*e*	AIC
LEM1a	Apteronotidae	Bioregion SS	82	7	DEC	− 329	2	0.022	0.013	662
LEM1b	Apteronotidae	Bioregion 2TS	82	7	DEC	− 288	2	0.056	0.026	580
LEM2a	Navajini	Bioregion SS	26	6	DEC	− 111	2	0.049	0.039	226
LEM2b	Navajini	Bioregion 2TS	26	6	DEC	− 106	2	0.082	0.056	216
LEM3a	Apteronotini	Bioregion SS	25	7	DEC	− 71	2	0.009	0.005	146
LEM3b	Apteronotini	Bioregion 2TS	25	7	DEC	− 66	2	0.028	0.009	136

Each biogeographical scenario represents a unique combination of three LEMs incorporating megariver captures in the Sub-Andean foreland and the dispersal-extinction-cladogenesis (DEC) model of geographic range evolution. Model fit as log-likelihood (lnL) values was assessed using the number of macroevolutionary parameters (K) and the Akaike Information Criterion (AIC). Bolded LEM favored by AIC. Abbreviations: GRE, general-rate-estimation; K, number of parameters; δ , macroevolutionary dispersal rate; e, extinction rate. Navajini (26 species) and Apteronotini (24 species) used to test alternative river capture models.

LEM1b estimates two origins of apteronotid species and clades in the Negro during the Early to Middle Miocene (c. 22 – 12 Ma) along the margins of the Caribbean-draining Pebas megawetland system, and 22 dispersal events into adjacent bioregions after the formation of the modern Atlantic-draining transcontinental Amazon river during the Late Miocene (c. 10 Ma; 10, 31, 33). In contrast, the one-time step stepping-stone paleogeographic model (LEM1a) estimates origins of Navajini in the Western Amazon before the formation of the modern Amazon river (Fig. S3). The two-time-slice LEM1b model also estimated origins of Sternarchorhynchini in rivers of the Brazilian Shield while the stepping-stone (LEM1a) model estimated origins of this clade in a westward-draining Rio Negro basin before c. 10 Ma.

Separate AAEs performed for the apteronotid tribes Navajini and Apteronotini also found best fits to the two time-slice models (LEM2 and LEM3b), which is consistent with predictions of the RCH (Figs. S4B, S5B). These LEMs model the effects of rare but high-impact Late Neogene megariver capture events on the formation of the diversity and distribution of these two apteronotid tribes.

## Discussion

### Drivers of apteronotid diversification

The results of this study provide empirical examples supporting all three of the hypotheses evaluated on the roles of rivers as drivers of Amazonian biodiversity and biogeography^8,34^. Because these hypotheses are not exclusive, they do not sum to 100%, making overlapping predictions regarding patterns of *alpha* and *beta* diversity among river basins (or interfluves), stream orders along the river continuum, and vicariance and dispersal events among large river basins through time.

The RBH is consistent with 46% of nodes and 47% of species in the phylogeny (Fig. S6, Tables S6 and S7). Prominent examples include sister-species pairs geographically isolated across the structural arches of the Sub-Andean Foreland (e.g., *Sternarchella calhamazon* and *S. patriciae*; *Sternarchella sima* and *S. orinoco*), Andean cordilleras (e.g., *Apteronotus cuchillejo* and *A. magoi*; *A. cuchillo* and *A. magdalenensis*), and lowland portals (e.g., *A. bonapartii* and *P. gimbeli*; *C. iara* and *C. compsa*; Fig. S7)^10,35^. Among the 60 terminal sister clades (Fig. 2A), 28 (43%) are allopatrically distributed among large river basins consistent with the IBH, and among the interfluves separating large river basins, consistent with the RBH.

The RNH is consistent with 15% of geographic distributions among apteronotid species (See SI text, Table S7). The RNH is indicated by higher *alpha* diversity in large rivers (SO 6-10) as assessed by species density, and higher *beta* diversity in smaller more fragmented rivers and streams (SO 1–5). These contrasting effects of *alpha* and *beta* diversity are reflected in divergence patterns between major clades, such as Navajini and Apteronotini, which exhibit ecological specializations to distinct stream-order environments, including habitat complexity in relation to flow velocity and substrate structure^36^. Deep river channels harbor surprisingly high species richness, likely due to high habitat volume, habitat heterogeneity, and geomorphic features of high-discharge systems^10,11,26^. These patterns follow well-established hydrological scaling rules (e.g., Horton’s Law) and suggest consistent relationships between stream size, habitat availability, and biodiversity^27,37^.

The RCH is consistent with 31% of nodes and 57% of species in the phylogeny (Fig. S6, Tables S6 and S7), reflecting signatures of major river reorganizations^23,32^. These include three mega river capture events: Eastern Amazon capturing parts of the Western Amazon [~10 Ma; 31], the Upper Negro River capturing the Upper Proto-Berbice (~7 Ma), and the Upper Paraguay River capturing part of the Upper Madeira basin [~4 Ma; 32], as well as four vicariance events linked to the uplift of the northern Andes^35^. These processes align with predictions of the RCH for species dispersal and range expansion across interfluvial barriers by the formation of new riverine corridors. Several taxa exhibit distributions consistent with past hydrological connections among large river basins through biogeographic portals, such as the Rupununi and Izozog wetlands and the Casiquiare Canal (Fig. S7). Such events enabled clades like the APS group to disperse outside the Amazon basin to the La Plata and Essequibo basins. Smaller river capture appears to have been efficacious in allowing several lineages of Apteronotini to expand their ranges to previously disconnected watersheds of the La Plata, Guiana-coastal, and trans-Andean Maracaibo, Magdalena and Pacific slope basins.

### Diversification of amazonian biotas

Results of this study highlight the complementary roles of environmental factors (e.g., riverscape dynamics) and organismal traits associated with habitat utilization, as primary drivers of Amazonian aquatic biodiversity^8,33,34^. Data supporting the RBH and IBH demonstrate that rivers serve as both corridors and barriers to organismal dispersal over a range of spatial and temporal scales, exposing populations to habitat heterogeneity among waterways with different stream orders and water chemistry profiles^12,34^. These environmental factors can in turn affect genetic isolation and ecological specialization among areas and habitats, ultimately affecting rates of speciation and extinction^6,38^.

The strong phylogenetic structuring of apteronotid clades, with highly distinct faunas in larger and smaller rivers, supports the RNH that stream order acts as a major axis of ecological and spatial differentiation^9,39^, structuring the species composition of local assemblages and guiding lineage diversification at a regional (basin-wide) scale^5,34^. The RNH is also consistent with a broader bimodal pattern of freshwater biodiversity across tropical South America, with high connectivity in lowland rivers and the high fragmentation in headwater streams. Among apteronotids, large rivers (SO 6–10) support greater alpha diversity, particularly in mainstems (SO 9–10; Fig. 3A, blue line), likely due to large habitat volumes enabling the coexistence of sympatric species^40^. Though representing only ~3% of river length, these channels contain ~91% of water volume and support dense, species-rich communities. Such patterns align with global biodiversity–area relationships but are uniquely amplified by Amazonia’s hydrological complexity [8, 12, 33; See SI text]. In contrast, smaller streams (SO 1–5), which dominate the Amazon’s linear network, support lower alpha but higher beta diversity. These habitats are ecologically heterogeneous and geographically isolated, promoting endemism and ecological specialization especially in taxa with narrow tolerances or limited dispersal. These findings align with global trends in fish, amphibian, aquatic invertebrate, and plant diversity, where species richness correlates with habitat size and net primary productivity^9^. Such differentiation contributes to the fine-scale structuring of diversity in fishes and supports the broader pattern of continental radiations occurring within patchworks of specialized microhabitats^28^. This dual pattern, where large rivers enable gene flow while small rivers drive isolation, underpins much of the region’s diversification and parallels trends in other major freshwater groups such as characiforms and cichlids^27,41^.

Data from this study also support the RCH that the long-term evolution of Amazonian biotas has also been strongly shaped by the dynamic, reticulated nature of river networks^10,25^. Tectonic activity, sediment accumulation, and changes in base level due to Andean uplift have repeatedly reconfigured drainage patterns, producing transient but biologically significant corridors and barriers among major river basins. Events like the formation of the Pebas system, interfluvial arch uplift, and marine incursions have fragmented and reconnected freshwater habitats, fueling pulses of dispersal and allopatric speciation in clades beyond fishes including frogs, reptiles, and floodplain-adapted plants^42^ (Table S1).

River capture, stochastically more common in lowland than upland tributaries, exemplifies this reticulation by enabling episodic faunal exchanges among drainages such as the Amazon, Orinoco, Essequibo, and La Plata. These connections often occur via lowland biogeographic portals, such as the Rupununi and Izozog wetlands and the Casiquiare Canal. The biogeographic history of Apteronotidae illustrates all these dynamics, with clades of deep-channel adapted species largely restricted to riverine corridors of large rivers, and clades of small-river and stream adapted species dispersing across watershed boundaries by means of river captures. These processes mirror broader patterns of Amazonian taxa, where repeated episodes of basin connectivity and fragmentation have driven the deep and complex biogeographic structure in other clades of fishes^10,19,32^, birds^12^, frogs^14^, mammals^16^, and plants^33^.

### Evolutionary perspective on conservation

The rapid pace of anthropogenic change in the Amazon Basin underscores the urgent need to understand how riverscape alterations influence biodiversity^43^. Human-driven modifications such as deforestation, dam construction, and river diversions are reshaping hydrological regimes at rates far exceeding natural processes^44^. These disruptions fragment habitats, alter sediment transport, and impede species dispersal, with long-term consequences for population structure and gene flow (See SI text). Because biodiversity formation occurs over timescales of thousands to millions of years, Amazonian ecosystems will not recover quickly on human or ecological time scales of decades to centuries^45^. Habitats and organismal ecophenotypic traits develop over evolutionary time, shaping how species respond to environmental changes. For instance, the isolation of aquatic assemblages due to habitat fragmentation presents a critical challenge. The construction of large river dams, such as the Tucuruí and Belo Monte dams on the Lower Tocantins and Xingu rivers (completed in 1984 and 2016, respectively), have drastically altered riverine ecosystems by impeding fish migrations and modifying environmental flow regimes^46^.

Evolutionary evidence from apteronotid knifefishes suggests that species’ persistence depends significantly on their ecophysiological traits. Rapids-adapted species, which rely on fast-flowing oxygen-rich waters, are at greater risk of decline compared to their non-rapids counterparts that exhibit broader physiological tolerances^47^. Similar patterns have been observed in other river systems; for example, the Three Gorges Dam on the Yangtze River has led to the decline of riverine species such as the Chinese paddlefish (*Psephurus gladius*), which was declared extinct in 2022 due to habitat fragmentation and disrupted migratory pathways^2^. These cases highlight the importance of a deep-time evolutionary perspective in developing conservation strategies for riverine taxa, incorporating clade-specific adaptations and the effects of rare but impactful ecosystem changes over geological time scales.

## Materials and methods

### Experimental design

River data were obtained from HydroSHEDS v1 (HydroBASINS and HydroRivers), focusing on major South American basins (e.g., Amazon, Orinoco, Paraná-Paraguay)^48^. The hybas_sa_lev03 dataset was used to subset 13 river basins. Strahler Stream orders (SO 1–10) were extracted in ArcMap, and total stream length (~ 5.3 million km), surface area (~ 764,000 km^2^), and volume (~ 410 million km^3^) were calculated by stream order and basin^49^. These are conservative estimates, as the number and total length of just the first order intermittent rivers and ephemeral streams is at least 51–60% globally^50^. Channel widths were estimated using MERIT Hydro and validated in Google Earth. Geographic ranges were created through the direct use of occurrences. Coordinates were processed using an automated routine designed to detect and remove georeferencing errors, following the steps recommended by Robertson et al. (2016)^51^. To avoid pseudoreplication, duplicate coordinates were excluded. We then visually inspected species distributions to identify suspicious records, and removed occurrences that fell outside the known geographic ranges of apteronotids as documented in the literature (N = 1466; see Data Availability; See SI text). Stream order was determined for each record based on hydrological data layers analyzed in a GIS framework (See SI text; Table S4). Alpha, beta, and gamma diversity were calculated by stream order (Table S5), with Whittaker’s beta (${\beta }_{W}$) and absolute turnover (${\beta }_{A}$) used to assess species turnover and sharing across stream orders, accounting for sampling and rare taxa. Alpha diversity was normalized to account for sampling variation across stream order (1).

1 $$N = \left( {x - x_{\min } } \right)/\left( {x_{\max } - x_{\min } } \right)$$

Beta diversity was measured in two ways to account for both regional and local species diversity as well as the role rare species play in differentiating multiple communities (See SI text). Whittaker’s beta (${\beta }_{W}$) was calculated for each stream order (2). Gamma as represented here is the total species diversity of all stream orders combined and alpha is the mean species diversity within river segments of the same stream order (Table S6).

2 $$\beta_{W} = \gamma /\alpha$$

Absolute species turnover (${\beta }_{A}$) was calculated between each combination of stream orders (3). The shared number of species between multiple stream orders (c) is subtracted from the alpha diversity.

3 $$\beta_{A} = \left( {S_{1} - c} \right) + \left( {S_{2} - c} \right)$$

### Phylogeny estimation

The phylogeny used in AAE analyses (Fig. 2) of valid apteronotid species was estimated using published data^52^ augmented with 32 species phylogenetically placed manually using the software package Mesquite v3.7. Species were placed in a position of maximum parsimony using morphological data derived from cleared and stained specimens and microCT imaging (See SI text). The tree file with branching order and branch lengths is available at (10.5281/zenodo.8475).

### Macroevolutionary dispersal rates

Macroevolutionary dispersal rate (δ) estimation from BioGeoBEARS^53^.

### Ancestral area estimation

We used a model-fitting framework to evaluate the effects of river network structure and paleogeography on the evolutionary history of Apteronotid electric fishes. We conducted AAE in the R package *BioGeoBEARS*^53^ using maximum likelihood to estimate the fit of alternative LEMs, and AIC scores to evaluate model performance accounting for differing numbers of the free parameters (dispersal and extinction). Greater (less negative) AIC values indicate better-fitting models. AIC values were assessed for three pairs of LEMs differing in taxonomic richness and scope (i.e., family vs. tribe level). Alternative LEMs were designed to contrast the biogeographic effects of low-order (SO 1–5) versus high-order (SO 6–10) stream networks, and to evaluate how the scale of river capture events influenced diversification patterns. All analyses employed the Dispersal-Extinction-Cladogenesis (DEC) model of geographic range evolution and seven bioregions of tropical South America (Figs. 4, S3, S4, S5). Multiple analyses were conducted using alternative LEMs that varied in bioregion connectivity, number of time slices, and number of taxa. Species were scored by presence/absence in each bioregion using data in Table S9.

Two model frameworks were applied: a DEC stepping-stone model (LEM1a) and a two-time-slice paleogeographic model (LEM1b). A stepping-stone model allows dispersal between adjacent regions only using a binary adjacency matrix (Table S10). The time-stratified model has different dispersal matrices in two time periods (60 –10 Ma and 10–0 Ma) reflecting prominent shifts in South American drainage evolution, including the rise of the modern Amazon, Orinoco, and La Plata basins^19,31,32,54^. Ancestral ranges were visualized as pie charts at internal phylogenetic nodes. To test whether river captures of different magnitudes produce distinct biogeographic outcomes, we applied these LEMs separately to Navajini (LEM2a and LEM2b) and Apteronotini (LEM3a and LEM3b).

## Supplementary Information

Below is the link to the electronic supplementary material.

Supplementary Material 1

## Data Availability

All datasets and analysis code used in this study are archived in Zenodo and publicly available at 10.5281/zenodo.17518284. The Zenodo archive includes a static snapshot of the GitHub repository, containing raw data files, R scripts, and metadata required to reproduce the analyses.
