# Supplementary material for: Riverscape dynamics and habitat utilization structure evolutionary diversification in a clade of Amazonian electric fishes
Source: Sci Rep. 2025 Nov 27;15:42450. doi: 10.1038/s41598-025-26512-0 (PMC12660728; doi:10.1038/s41598-025-26512-0)
Supplement: Supplementary file 1 — Supplementary Material 1 [file 41598_2025_26512_MOESM1_ESM.docx]

Supplementary Materials for

**Riverscape dynamics and habitat utilization structure evolutionary diversification in a clade of Amazonian electric fishes**

*Jonathan Allen *et al.*

*Corresponding author. Email: jallen5368@gmail.com

**This file includes:**

Supplementary Text

Figs. S1 to S7

Tables S1 to S10

SI References (1 to 59)

Supplementary Text

GIS Stream Order Assignment

We assigned Strahler stream order (SO) values to each georeferenced species record using a GIS-based workflow in ArcMap. First, we generated stream order classifications from hydrological layers with the ArcMap Hydrology toolbox following the Strahler method. To associate occurrence points with particular stream orders, we created buffer zones around each stream segment, with buffer widths scaled relative to SO values following parameters established in previous literature [60]. Species occurrence coordinates were then snapped to the nearest buffer, ensuring consistent assignment to a stream order category. In cases where points fell near stream confluences or within low-resolution areas, stream order assignments were ambiguous. For these records, we performed manual validation using documented distribution ranges from the taxonomic and biogeographic literature to determine the most appropriate stream order. Final assignments for all records are reported in Table S4.

Experimental Design

To quantify patterns of beta diversity across stream orders, we employed two complementary approaches following the conceptual framework [1]. We calculated Whittaker’s beta diversity where γ is the total species richness across all river segments within a stream order, and α is the mean species richness across those individual river segments. This method was applied only to stream orders 5–6, as lower-order streams [1–4] lacked segments with sufficient statistical relevance. This index reflects the degree of compositional turnover among river segments of similar stream order. To evaluate species turnover between stream orders, we calculated the absolute number of unshared species where *S₁* and *S₂* are the species richness values for each of two stream orders, and *c* is the number of species shared between them. This approach captures the net species turnover across stream order boundaries and highlights how composition changes with increasing hydrological scale. We used Eschmeyer's *Catalog of Fishes* online database to validate species names and ensure taxonomic consistency across all phylogenetic and distributional datasets [2].

To evaluate support for the River Capture Hypothesis (RCH), we quantified the proportion of phylogenetic nodes showing evidence of river capture events. Nodes were considered influenced by RCH when their geographic distributions corresponded to historical drainage rearrangements documented in the literature. Evidence for RCH was calculated as the number of such nodes divided by the total number of nodes in the phylogeny (Fig. S6). For the River Barrier Hypothesis (RBH), we assessed all sister clade nodes to determine whether their distributions were allopatric and separated by major interfluvial regions. Support for RBH was calculated as the number of allopatrically distributed sister clade nodes divided by the total number of sister nodes.

In addition to node-based calculations, we evaluated evidence for the RCH, RBH, and RNH at the species level (Table S6). For RCH, we counted species whose present-day distributions are consistent with historical river capture events documented in the literature. The proportion of species affected by RCH was calculated as the number of species with evidence of river capture divided by the total number of species in the dataset. For RBH, we identified species whose sister taxa occur in allopatry across interfluvial regions. The proportion of species affected by RBH was calculated as the number of species with allopatric sister taxa divided by the total number of species with identifiable sister relationships. For RNH, we assessed habitat associations by stream order. Species were considered affected by RNH if their closest relatives occurred in different sets of stream orders, indicating diversification across riverine habitats of contrasting size and connectivity. The proportion of species affected by RNH was calculated as the number of such species divided by the total number of species in the dataset.

Phylogenetic Topology Modifications

We based our phylogenetic framework on the maximum likelihood topology published by [3]. To expand taxonomic coverage and reflect recent taxonomic and morphological data, we incorporated an additional 32 apteronotid species into this topology. These additions were placed using a maximum parsimony approach informed by morphological characters derived from cleared and stained specimens, as well as high-resolution microCT imaging [4, 5]. All topological modifications were conducted in the software *Mesquite* v3.61 [6].

When integrating new taxa or modifying relationships, halfway branches were adjusted to reflect approximate midpoint positions within clades based on morphological affinity. A major revision involved rendering the tribe Apteronotini monophyletic by nesting the *leptorhynchus* clade with the *albifrons* clade, consistent with phylogenetic hypotheses in [7]. We also repositioned *Apteronotus magoi* alongside *A. cuchillejo* based on the morphological similarities noted by [8], and included *Megadontognathus* following [9].

Within the tribe Navajini, we revised the placement of *Porotergus* spp., removing *“A.” ellisi* due to taxonomic uncertainty and lack of clear phylogenetic placement. Two species of *Sternarchella* (*S. sima* and *S. orinoco*) were added based on taxonomic revisions [10], and *Sternarchogiton zuanoni* was incorporated [11]

In the tribe Sternarchorhynchini, several additional species were included based on morphological analyses [12]. For the Adontosternarchini, *Adontosternarchus duartei* was added based on its recent description [13]. All phylogenetic placements reflect the most up-to-date hypotheses and available morphological evidence, providing a comprehensive framework for the biogeographic and ecological analyses presented in this study (see Data Availability).

Species Area Density

To evaluate species-area relationships (SAR) in Apteronotidae, we analyzed river segments where apteronotid diversity ranged from 5 to 10 species and where species richness (N) was greater than or equal to 1. Each river segment’s surface area (km²) was calculated, and species richness was plotted against segment area to assess scaling patterns. A power function trendline was fitted to the data to model the SAR using the equation *S = cA^z^*, where *S* is species richness, *A* is area, and *c* and *z* are fitted constants. This approach allows for quantifying how species richness scales with area across different hydrological environments [14].

Diversification of Amazonian Biotas

Although large rivers constitute a small fraction of total river length (~3%), they encompass the vast majority of water volume (~91%) and exert a disproportionate influence on regional biodiversity. Meanwhile, *terra firme* streams contribute outsized evolutionary importance due to their ecological isolation, high endemism, and potential for niche specialization seen in other South American freshwater fishes. These dual environments form the backbone of the region’s continental-scale radiations, where species diversify both within and between major hydrological compartments. These results reflect a broader continental-scale pattern of two distinct freshwater faunas, one in small rivers and streams and another in large rivers which is consistent with global bimodal distributions of riverine habitat area [Fig. 3D from 15].

Mainstem rivers of higher Strahler Stream Orders (SO 6–10) exhibit a notably higher baseline species richness, even at relatively small spatial scales, as indicated by elevated values of the species-area curve constant (C = 232,210). This pattern reflects intrinsic characteristics of these larger river habitats, including greater habitat volume, increased environmental heterogeneity, enhanced primary productivity, and higher degrees of connectivity, all of which collectively support richer species assemblages independent of area effects. The elevated C value in mainstem rivers implies that these systems function as disproportionately important reservoirs of biodiversity prior to considering species-area scaling effects (Table S8). From a conservation standpoint, the observed increase of C with stream order underscores the necessity of prioritizing both large rivers due to their extensive species-rich communities and small tributaries, which often sustain unique or endemic taxa despite exhibiting lower overall species richness.

Evolutionary perspective on conservation

Extensive deforestation in the Amazon Basin, driven primarily by agricultural expansion and logging, has led to habitat loss and fragmentation, disrupting connectivity for terrestrial and aquatic species [16, 17]. The proliferation of hydroelectric dams, with over 400 existing and many more planned, has fundamentally altered river hydrology, sediment transport, and fish migration routes, impacting population structure and gene flow [18, 19]. Models provide a powerful framework for predicting how these shifting landscapes and riverscapes shape diversification, offering insights that extend beyond present-day patterns to forecast future evolutionary trajectories. Given the unprecedented rates of habitat modification, deforestation, and river fragmentation, it is imperative to develop robust models that capture the dynamic interplay between landscape change and speciation, ensuring a more comprehensive understanding of biodiversity responses to anthropogenic pressures.


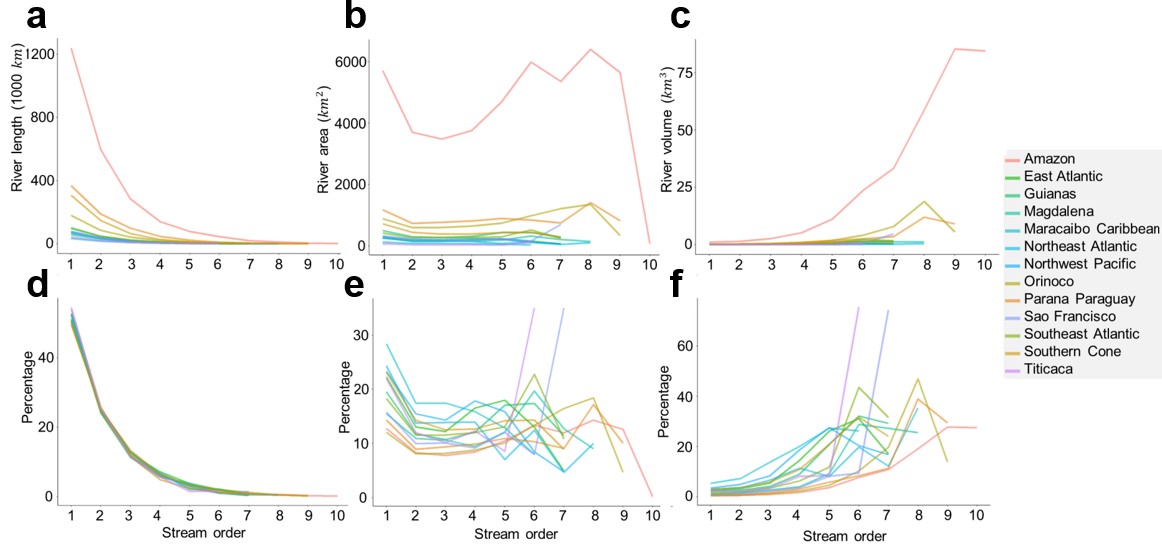


**Fig. S1. Habitat** **metrics for 13 large river basins of tropical South America.** Summed data for all river segments between tributaries arranged by Strahler stream order (SO) from volume. Note following Horton’s Laws of stream numbers and stream lengths (20) and most river length is headwater tributaries with SO1 and most river volume is the largest rivers SO9 and SO10 HydroSHEDS. **a.** Channel length (km). **b.** Surface area (km^2^). **c.** Water volume (thousand meters^3^). **d.** Proportion total river length. **e.** Proportion total river area. **f.** Proportion total river volume.


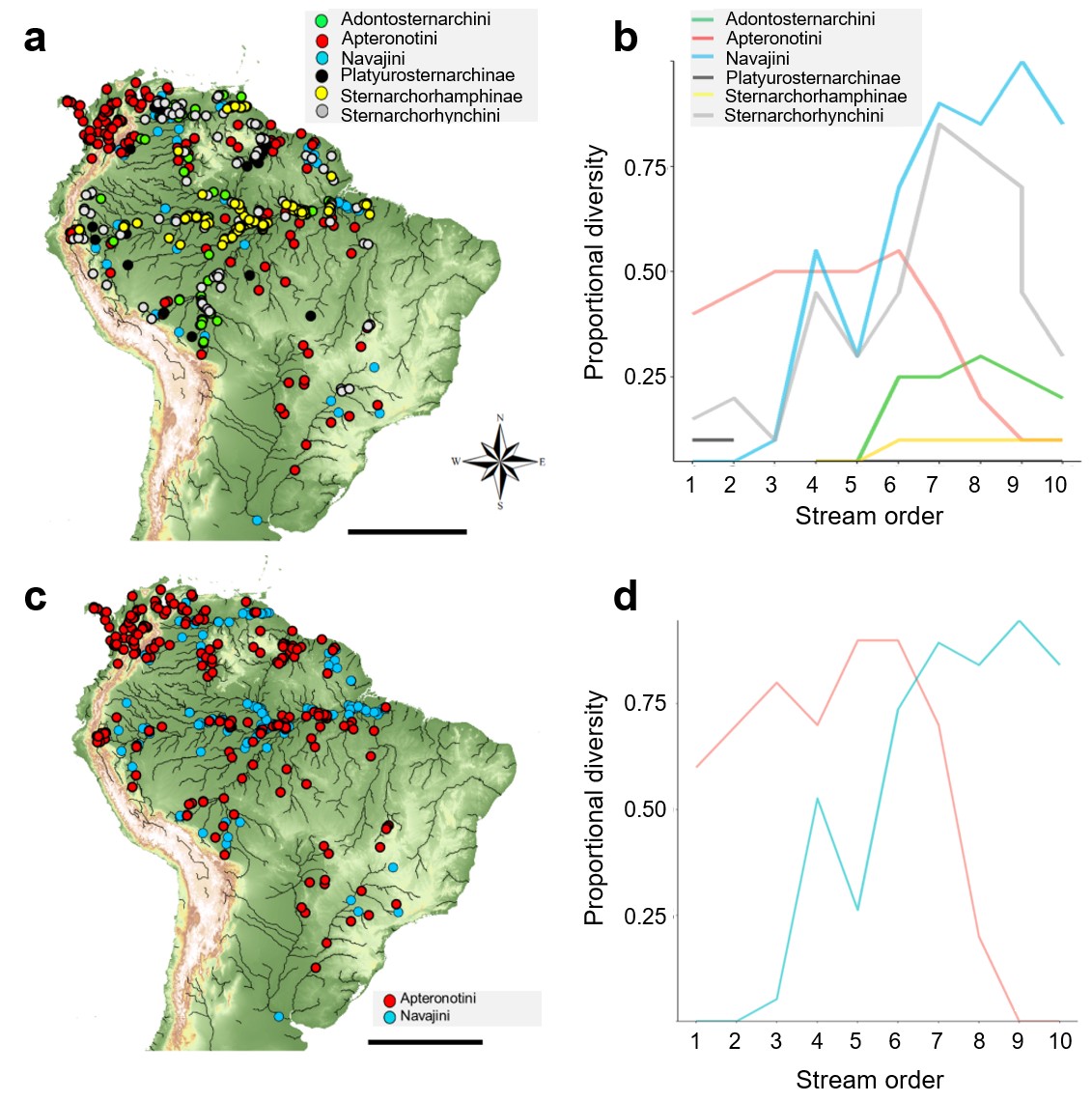


Fig. S2. Geographic distributions and proportional diversity by SO for six apteronotid clades. a. Six subclades of Apteronotidae distributed throughout South America. Scale bar = 1,000 km. b. Normalized diversity within each subclade by stream order. c. Distribution of the Apteronotini (red circles) and Navajini (blue circles). Scale bar = 1,000 km. d. Normalized diversity (species richness) of the two clades, Apteronotini and Navajini by stream order.


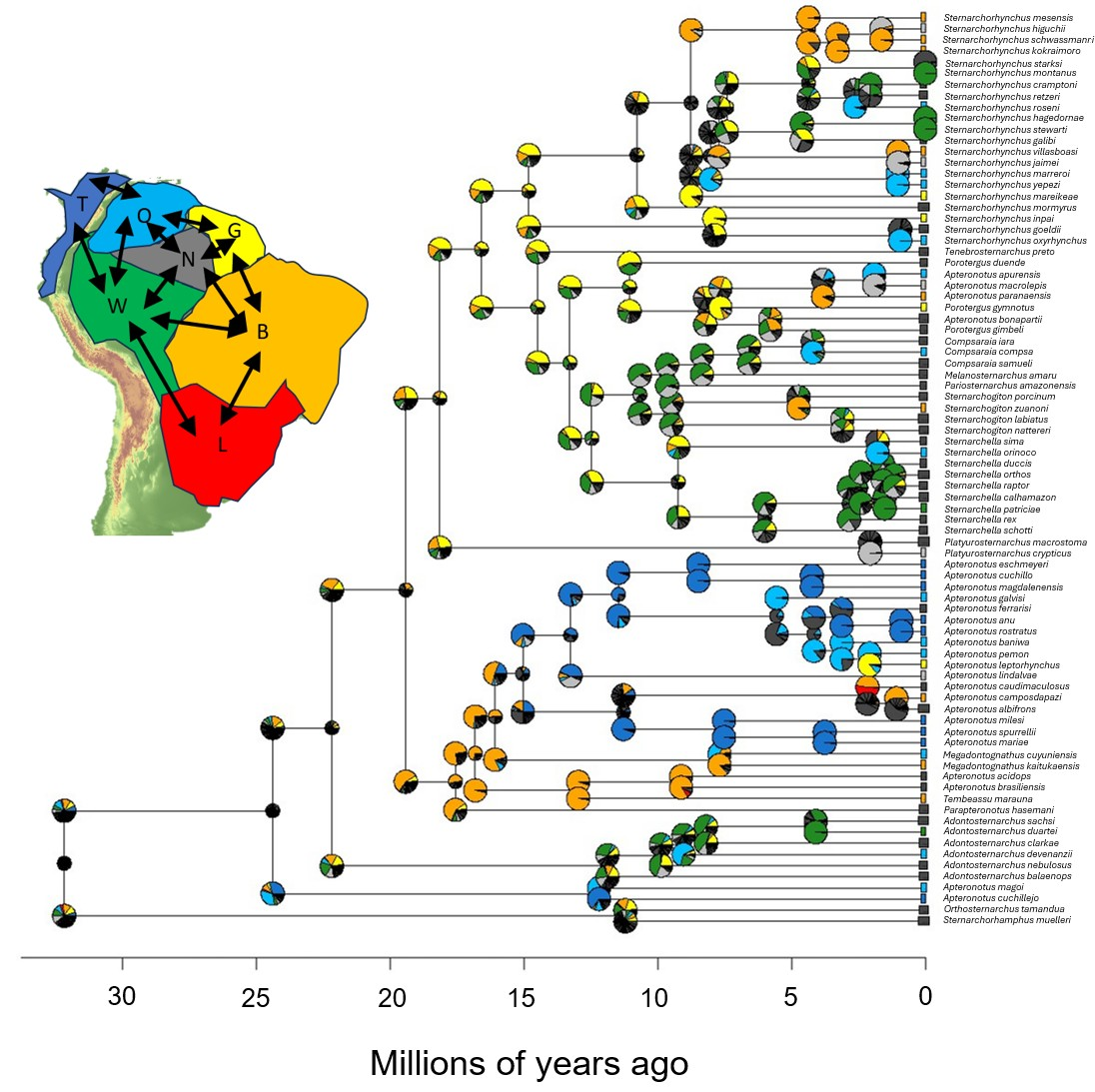


**Fig. S3.** **AAE of Apteronotidae using one-time step stepping-stone paleogeographic model (LEM1a).** Time-calibrated phylogeny from this study. Pie charts represent marginal likelihoods of ancestral areas using the DEC model in BioGeoBEARS. Stepping stone model of bioregion connectivity with unconstrained ancstates. Bioregions in inset; B = Brazilian Shield, G = Guiana Shield, L = La Plata, N = Negro, O = Orinoco, T = Trans-Andean, W = Western Amazon.

**
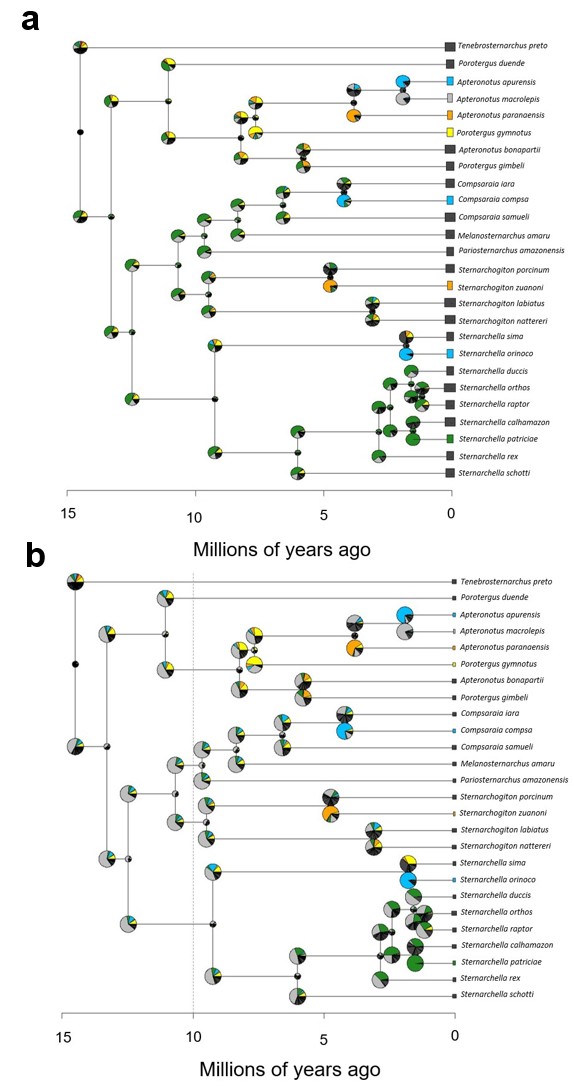
**

**Fig. S4. AAE for Navajini used assessing the RNH**. Methods and graphical conventions as in Fig. S3. **a.** Stepping stone model with unconstrained ancstates: global optim, six areas max. **b.** Two-step DEC model with unconstrained ancstates: global optim, seven areas max. Time slices from 50-10 Ma and 10-0 Ma.


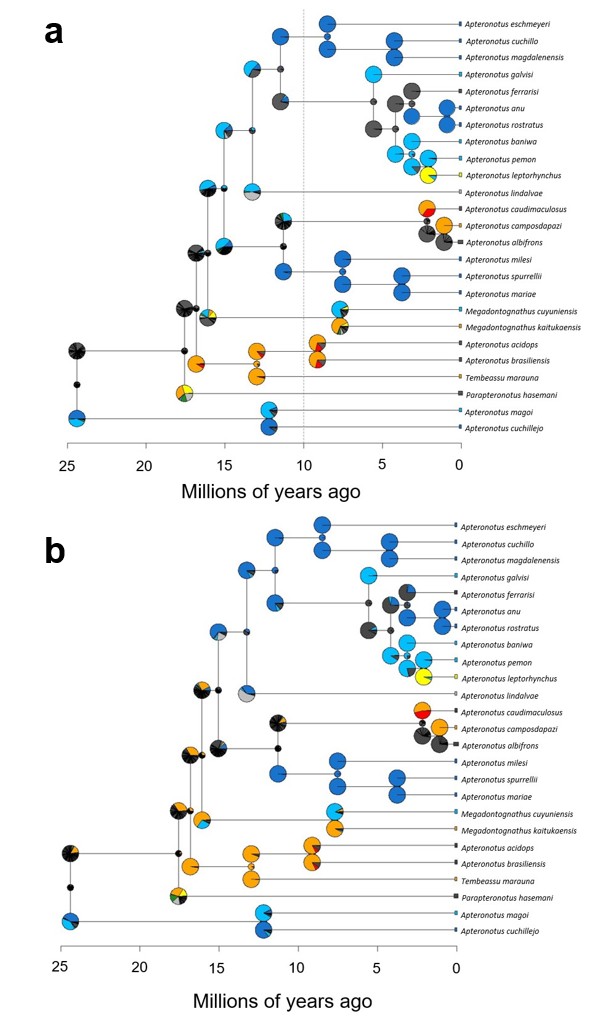


**Fig. S5. AEA for Apteronotini assessing the RNH**. Methods and graphical conventions as in Fig. S3. **a.** Stepping stone model with unconstrained ancstates: global optim, seven areas max. **b.** Two-step DEC model with unconstrained ancstates: global optim, seven areas max. Time slices from 25-10 Ma and 10-present.


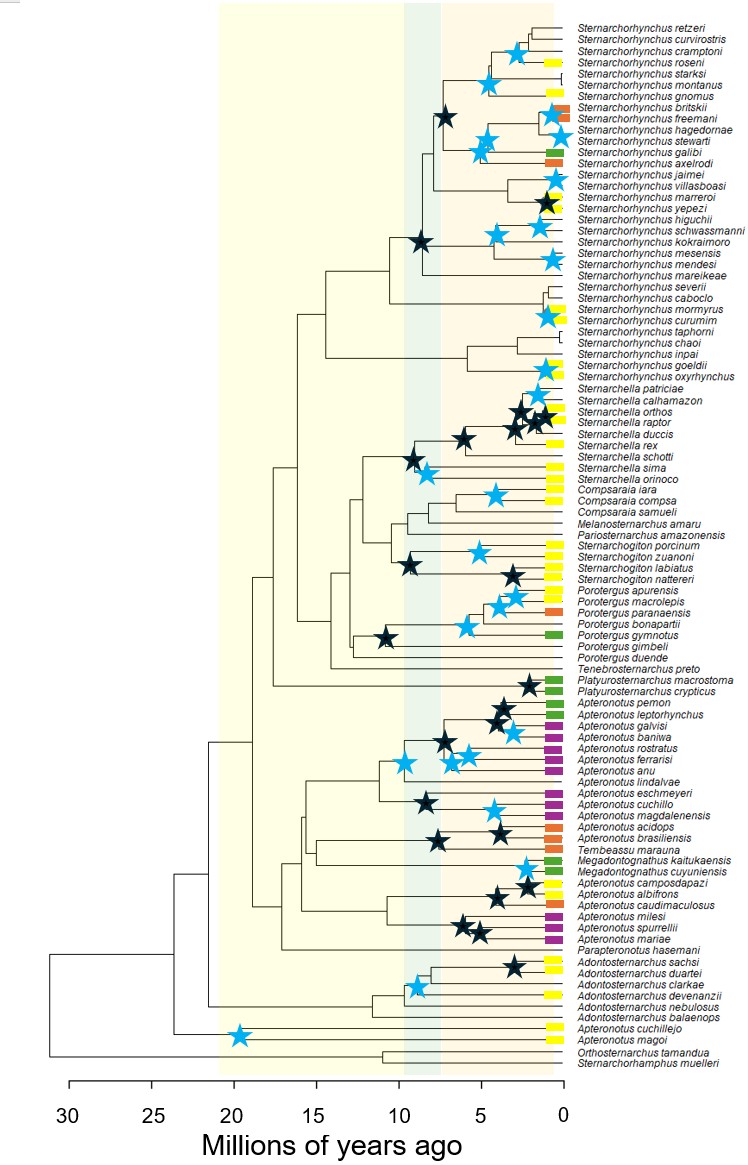


**Fig. S6. Phylogenetic evidence for diversification under RBH and RCH**. Stars mark clades inferred to be affected by river capture (51 of 163 nodes; 31%), with blue stars highlighting sister-clade pairs exhibiting allopatric distributions consistent with RBH (28 of 60 sister nodes; 46%). Colored terminal boxes indicate species associated with specific geographic events: Yellow = Eastern Amazon capturing Western Amazon c. 10 Ma ; Green = Negro capturing Upper ProtoBerbice c. 7 Ma ; Purple = Vicariance associated with Northern Andes; Orange = Paraguay capturing Upper Madeira c. 4 Ma.


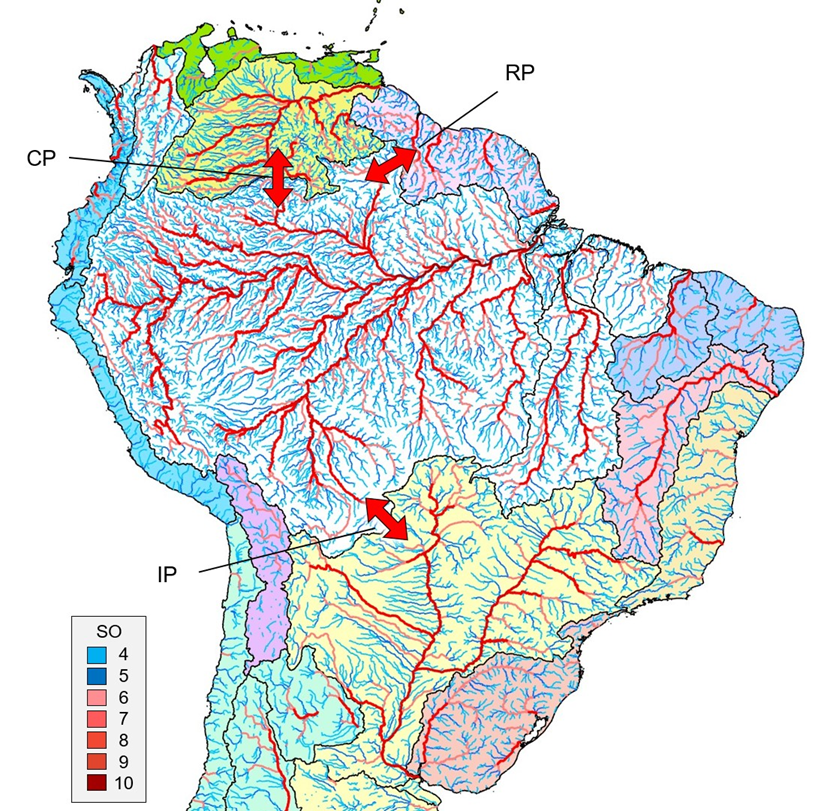


**Fig. S7. Major rivers and drainage basins of tropical South America.** Rivers by Strahler stream order (SO). Biogeographic portals between Amazonian tributaries and adjacent basins as double-headed arrows. CP, Casiquiare Portal; RP, Rupununi Portal; IP, Izozog Portal. Map data from HydroSHEDS.

**Table S1.** Published evidence for the influence of River Capture (RCH), Riverine Barrier (RBH), and River Network (RNH) hypotheses on lineage diversification across Amazonian and adjacent biotas. Entries are organized by major clade (e.g., fishes, birds, mammals, plants), with citations indicating empirical support for one or more of the three riverscape diversification hypotheses.

**Table S2.** Materials examined (number of specimens and species) broken up by subclade within the family Apteronotidae and organized by analyses. Indet = “*Apteronotus*” *magoi* and “*A*.” *cuchillejo*.

**Table S3.** Adult modal habitat utilization of 96 apteronotid species. Habitats: DC = Deep Channels, RM = River Margins, RR = Riffles and Rapids, TF = *Terra Firme* streams. Taxa arranged by alphabetically taxonomic tribe and genus. R# = reference (see supplemental bibliography).

**Table S4.** Occurrence data of 96 apteronotid species by Stream Order (SO). SO1, headwater streams; SO10 = largest river (i.e., lower Amazon). *, no records. Data representing 1466 georeferenced localities on (see Data Availability).

**Table S5.** River segments by SO for diversity analyses reported in Figure 3. Stream orders 1-4 excluded due to stochastic sampling effects. Note: the lower Amazon is the only waterway of SO 10.

**Table S6.** Empirical examples of RBH and RCH among apteronotids. Note: RBH nodes are sister clades with unambiguous ancestral area estimation in Fig. 4 RCH nodes are estimated from Figs 2 & S6. For taxa with disjunct geographic distributions among basins and estimated node ages coinciding with published dates for river capture events.

**Table S7.** Evidence for three diversification hypotheses in apteronotid species. RBH, indicated by allopatric distributions between sister species; RNH, indicated by distributions across differing stream orders; RCH, indicated by evidence shown in Fig. see Figs. 4 & S6. Presence of evidence is denoted by an ‘x’ in each category.

**Table S8.** Species density estimates for apteronotids among subbasins partitioned by river size. Subbasins from HydroSHEDS. Species density as C = S/A^z^ [14].

**Table S9.** Occurrence data for 82 Apteronotidae species across seven biogeographic regions. Species are assigned to separate analyses based on their inclusion in different LEMs: **LEM1** (Apteronotidae), LEM2 (Navajini), and LEM3 (Apteronotini). These classifications reflect regional species distributions and their corresponding biogeographic histories.

**Table S10.** Landscape Evolution Models (LEMs) used in the stepping-stone (a) and two-time-slice (b) analyses, each incorporating different taxon sampling. LEM1 represents the full dataset including all Apteronotidae species, LEM2 focuses on the Navajini clade, and LEM3 examines the Apteronotini clade. These models account for lineage-specific biogeographic histories and dispersal dynamics across riverine landscapes.

**SI References**

1. Tuomisto, H., A diversity of beta diversities: straightening up a concept gone awry. Part 1. Defining beta diversity as a function of alpha and gamma diversity. *Ecography* **33**, 2–22 (2010).
2. Fricke, R., Eschmeyer, W. N., van der Laan, R., (eds), Eschmeyer’s catalog of fishes: genera, species, references (http://researcharchive.calacademy.org/research/ichthyology/catalog/fishcatmain.asp). Electronic version accessed 21 May (2025).
3. Tagliacollo, V. A., et al., Time-calibrated phylogeny of neotropical freshwater fishes. *Front. Bioinform.* **4**, 1433995 (2024).
4. Albert, J. S., Crampton, W. G. R., A new species of electric knifefish, genus *Compsaraia* (Gymnotiformes: Apteronotidae) from the Amazon River, with extreme sexual dimorphism in snout and jaw length. *Syst. Biodivers.* **7**, 81–92 (2009).
5. Ford, K. L., Bernt, M. J., Summers, A. P., Albert, J. S., Mosaic evolution of craniofacial morphologies in ghost electric fishes (Gymnotiformes: Apteronotidae). *Ichthyol. Herpetol.* **110**(2), 315–326 (2022).
6. Maddison, W. P., Maddison, D. R., *Mesquite: A modular system for evolutionary analysis*. Version 2.0 (2007). Available at: http://www.mesquiteproject.org
7. Bernt, M. J., Tagliacollo, V. A., Albert, J. S., Molecular phylogeny of the ghost knifefishes (Gymnotiformes: Apteronotidae). *Mol. Phylogenet. Evol.* **135**, 297–307 (2019).
8. De Santana, C. D., Castillo, O., Taphorn, D., *Apteronotus magoi*, a new species of ghost knifefish from the Río Orinoco basin, Venezuela (Gymnotiformes: Apteronotidae). *Ichthyol. Explor. Freshw.* **17**(3), 275–280 (2006).
9. Campos-da-Paz, R., New species of *Megadontognathus* from the Amazon basin, with phylogenetic and taxonomic discussions on the genus (Gymnotiformes: Apteronotidae). *Copeia*, 1041–1049 (1999).
10. Evans, K. M., Crampton, W. G. R., Albert, J. S., Taxonomic revision of the deep channel electric fish genus Sternarchella (Teleostei: Gymnotiformes: Apteronotidae), with descriptions of two new species. *Neotrop. Ichthyol.* **15** (2017).
11. De Santana, C. D., Vari, R. P., New rheophilic species of electric knifefish from the rapids and waterfalls of the lower Rio Xingu, Brazil (Gymnotiformes: Apteronotidae). *Copeia*, **1**, 160–164 (2010).
12. De Santana, C. D., Vari, R. P., Electric fishes of the genus *Sternarchorhynchus* (Teleostei, Ostariophysi, Gymnotiformes); phylogenetic and revisionary studies. *Zool. J. Linn. Soc.* **159**(1), 223–371 (2010).
13. Duarte, C., Espírito-Santo, H. M. V., Zuanon, J., Py-Daniel, L. R., Deus, C. P., What happens in the darkness? Seasonal variations in tropical benthic fish assemblages. *Mar. Freshw. Res.* **71**(4), 419–431 (2019).
14. Rosenzweig, M. L., Applying species–area relationships to the conservation of species diversity, in Frontiers of Biogeography: New Directions in the Geography of Nature, Lomolino, M. V., Heaney, L. R., Eds. (Sinauer Associates, Sunderland, MA, 2004), pp. 325–343.
15. He, C., et al., A global dataset of the shape of drainage systems. *Earth Syst. Sci. Data* **16**, 1151–1166 (2024).
16. Flores, B. M., et al., Critical transitions in the Amazon forest system. *Nature* **626**, 555–564 (2024).
17. Barlow, J., et al., Anthropogenic disturbance in tropical forests can double biodiversity loss from deforestation. *Nature* **535**, 144–147 (2016).
18. Winemiller, K. O., et al., Balancing hydropower and biodiversity in the Amazon, Congo, and Mekong. *Science* **351**, 128–129 (2016).
19. Latrubesse, E. M., et al., Damming the rivers of the Amazon basin. *Nature* **546**, 363–369 (2017).
20. Shreve, R. L., Statistical law of stream numbers. *J. Geol.* **74**(1), 17-37 (1966).
21. Londoño-Burbano, A., Britto, M. R., Species delimitation and historical biogeography of *Sturisoma* Swainson, 1838 (Loricariidae: Loricariinae): Hidden diversity along the Amazon River. *Mol. Phylogenet. Evol.* **203**, 108248 (2025).
22. Kim, L. Y., Crampton, W. G., Albert, J. S., Two new species of *Gymnotus* (Gymnotiformes: Gymnotidae) from Brazil and historical biogeography of the subgenus *Lamontianus*. *Copeia* **108**, 468–484 (2020).
23. Waters, J. M., Burridge, C. P., Craw, D., River capture and freshwater biological evolution: a review of galaxiid fish vicariance. *Diversity*, **12**(6), 216 (2020).
24. Godinho, M. B. C., Da Silva, F. R., The influence of riverine barriers, climate, and topography on the biogeographic regionalization of Amazonian anurans. *Sci. Rep.* **8**, 3427 (2018).
25. Moraes, L. J., Pavan, D., Barros, M. C., Ribas, C. C., The combined influence of riverine barriers and flooding gradients on biogeographical patterns for amphibians and squamates in south-eastern Amazonia. *J. Biogeogr.* **43**, 2113–2124 (2016).
26. Fouquet, A., et al., The trans-riverine genetic structure of 28 Amazonian frog species is dependent on life history. *J. Trop. Ecol.* **31**, 361–373 (2015).
27. Klabacka, R. L., et al., Rivers of Indochina as potential drivers of lineage diversification in the spotted flying lizard (*Draco maculatus*) species complex. *Mol. Phylogenet. Evol.* **150**, 106861 (2020).
28. Pirani, R. M., et al., Testing main Amazonian rivers as barriers across time and space within widespread taxa. *J. Biogeogr.* **46**(11), 2444–2456 (2019).
29. Musher, L. J., et al., M. Giakoumis, J. Albert, G. Del-Rio, M. Rego, G. Thom, J. Cracraft, River network rearrangements promote speciation in lowland Amazonian birds. *Sci. Adv.* **8**, eabn1099 (2022).
30. Naka, L. N., Costa, B. M. D. S., Lima, G. R., Claramunt, S., Riverine barriers as obstacles to dispersal in Amazonian birds. *Front. Ecol.. Evol.* **10**, 846975 (2022).
31. Ribas, C. C., Aleixo, A., Nogueira, A. C., Miyaki, C. Y., Cracraft, J., A palaeobiogeographic model for biotic diversification within Amazonia over the past three million years. *Proc. R. Soc. B* **279**, 681–689 (2012).
32. Aleixo, A., Historical diversification of a terra‐firme forest bird superspecies: a phylogeographic perspective on the role of different hypotheses of Amazonian diversification. *Evolution*, **58**(6), 1303–1317 (2004).
33. Janiak, M. C., et al., Two hundred and five newly assembled mitogenomes provide mixed evidence for rivers as drivers of speciation for Amazonian primates. *Mol. Ecol.* **31**(14), 3888–3902 (2022).
34. Boubli, J. P., et al., Spatial and temporal patterns of diversification on the Amazon: A test of the riverine hypothesis for all diurnal primates of Rio Negro and Rio Branco in Brazil. *Mol. Phylogenet. Evol.* **82**, 400–412 (2015).
35. Byrne, M. S., Quintana, R. D., Bolkovic, M. L., Cassini, M. H., Túnez, J. I., The role of river drainages in shaping the genetic structure of capybara populations. *Genetica*. **143**, 645–656 (2015).
36. Anthony, N. M., et al., The role of Pleistocene refugia and rivers in shaping gorilla genetic diversity in central Africa. *PNAS* **104**, 20432–20436 (2007).
37. Householder, J. E., et al., One sixth of Amazonian tree diversity is dependent on river floodplains. *Nat. Ecol. Evol.* **8**(5), 901–911 (2024).
38. Wu, L. X., et al., Rivers have shaped the phylogeography of a narrowly distributed cycad lineage in Southwest China. *Conserv. Genet.* **25**(2), 439–453 (2024).
39. Nazareno, A. G., Dick, C. W., Lohmann, L. G., Wide but not impermeable: Testing the riverine barrier hypothesis for an Amazonian plant species. *Mol. Ecol*. **26**(14), 3636–3648 (2017).
40. Triques, M. L., *Apteronotus acidops*, new species of long-snouted electric fish (Teleostei: Gymnotiformes: Apteronotidae) from the upper rio Paraná basin in Brazil, with a key to the apteronotid species from the area. *Vertebr. Zool*. **61**(3), 299–306 (2011).
41. Crampton, W. G. R., Effects of anoxia on the distribution, respiratory strategies and electric signal diversity of gymnotiform fishes. *J. Fish Biol.* **53**, 307–330 (1998).
42. De Santana, C. D., Vari, R. P., Brown ghost electric fishes of the *Apteronotus leptorhynchus* species-group (Ostariophysi, Gymnotiformes); monophyly, major clades, and revision. *Zool. J. Linn. Soc.* **168**, 564–596 (2013).
43. De Santana, C. D., Lehmann, P. A., *Apteronotus camposdapazi*, a new species of black ghost electric knifefish, from the Rio Tocantins basin, Brazil (Gymnotiformes: Apteronotidae). *Ichthyol. Explor. Freshw.* **17**(3), 261–266 (2006).
44. De Santana, C. D., *Apteronotus caudimaculosus* n. sp. (Gymnotiformes: Apteronotidae), a sexually dimorphic black ghost knifefish from the Pantanal, western Brazil, with a note on the monophyly of the *A. albifrons* species complex. *Zootaxa* **252**(1), 1–11 (2003).
45. Maldonado-Ocampo, J. A., Vari, R. P., Usma, J. S., Checklist of the freshwater fishes of Colombia. *Biota Colombiana* **9**(2), 143–237 (2008).
46. Maldonado-Ocampo, J. A., de Santana, C. D., New records of *Apteronotus eschmeyeri* (Gymnotiformes: Apteronotidae) in the Magdalena-Cauca hydrographic region, Colombia. *Dahlia*. **8**, 25–27 (2005).
47. De Santana, C. D., Maldonado-Ocampo, J. A., Redescription of *Apteronotus mariae* (Eigenmann, Fisher, 1914) and the taxonomic status of *Apteronotus jurubidae* (Fowler,1944) (Gymnotiformes: Apteronotidae). *Zootaxa* **632**(1), 1–14 (2004).
48. De Santana, C. D., Fernandes, C. C., A new species of sexually dimorphic electric knifefish from the Amazon Basin, Brazil (Gymnotiformes: Apteronotidae). *Copeia*, **2**, 283–292 (2012).
49. Maldonado-Ocampo, J. A., de Santana, C. D., Crampton, W. G. R., On *Apteronotus magdalenensis* (Miles, 1945) (Gymnotiformes: Apteronotidae): a poorly known species endemic to the río Magdalena basin, Colombia. *Neotrop. Ichthyol.* **9**(3), 505–514 (2011).
50. De Santana, C. D., Maldonado-Ocampo, J. A., *Apteronotus milesi*, new species of ghost knifefish (Gymnotiformes: Apteronotidae) from the Cauca River, with a key to apteronotids from the Magdalena-Cauca basin, Colombia. *Ichthyol Explor. Freshw.* **16**(3), 223–230 (2005).
51. Fernandes, C. C., Lundberg, J. G., Riginos, C., Largest of all electric-fishes snouts: hypermorphic facial growth in male *Apteronotus hasemani* and the identity of *Apteronotus anas* (Gymnotiformes: Apteronotidae). *Copeia*, 52–61 (2002).
52. M. Sabaj pers. comm.
53. Crampton, W. G., Diversity and adaptation in deep channel Neotropical electric fishes. In: P. Sebert, D. W. Onyango, B. G. Kapoor, editors. Fish life in special environments. Enfield (NH): Science Publishers. p. 283–339 (2007).
54. Bernt, M. J., Albert, J. S., A New Species of Deep-channel Electric Knifefish *Compsaraia* (Apteronotidae, Gymnotiformes) from the Amazon River. *Copeia* **105**(2), 211–219 (2017).
55. Albert, J. S., Crampton, W. G. R., A new genus and species of Neotropical electric fish (Gymnotiformes: Apteronotidae) from the Amazon River. *Ichthyol. Explor. Freshw.* **17**, 267–274 (2006).
56. De Santana, C. D., Crampton, W. G. R., A review of the South American electric fish genus *Porotergus* (Gymnotiformes: Apteronotidae) with the description of a new species. *Copeia*, **1**, 165–175 (2010).
57. Lundberg, J. G., Fernandes, C. C., Albert, J. S., Garcia, M., *Magosternarchus*, a new genus with two new species of electric fishes (Gymnotiformes: Apteronotidae) from the Amazon River Basin, South America. *Copeia*. **3**, 657–670 (1996).
58. De Santana, C. D., Vari, R. P., The South American electric fish genus *Platyurosternarchus* (Gymnotiformes: Apteronotidae). *Copeia*, **2**, 233–244 (2009).
59. Triques, M. L., *Tembeassu marauna*, new genus and species of electrogenic neotropical fish (Ostariophysi: Gymnotiformes: Apteronotidae). Revue française d'Aquariologie Herpétologie. **25**, 5–10 (1998).
60. Downing, J. A., Cole, J. J., Duarte, C. M., Middelburg, J. J., Melack, J. M., Prairie, Y. T., Kortelainen, P., Striegl, R. G., McDowell, W. H., and Tranvik, L. J., Global abundance and size distribution of streams and rivers. *Inland Waters*, **2**(4), 229–236.
